# Do statins decrease testosterone in men? Systematic review and meta-analysis

**DOI:** 10.1590/S1677-5538.IBJU.2023.0578

**Published:** 2024-03-18

**Authors:** Felipe Placco Araujo Glina, Leonardo Lopes, Rodrigo Spinola e Silva, Eduardo Augusto Correa Barros, Bruno Biselli, Sidney Glina

**Affiliations:** 1 Faculdade de Medicina do ABC Disciplina de Urologia Santo André SP Brasil Disciplina de Urologia, Faculdade de Medicina do ABC, Santo André, SP, Brasil;; 2 Universidade de São Paulo Hospital das Clinicas Instituto do Coração São Paulo SP Brasil Departamento de Insuficiência Cardíaca, Instituto do Coração (InCor) - Hospital das Clinicas, Universidade de São Paulo - USP, São Paulo, SP, Brasil

**Keywords:** Testosterone, 5-alpha Reductase Inhibitors, Meta-Analysis [Publication Type]

## Abstract

**Purpose:**

Statins are one of the most prescribed classes of drugs worldwide to treat hypercholesterolemia and dyslipidemia. By lowering the level of cholesterol, the use of statin could cause a reduction in testosterone levels.

The objective was to evaluate whether the continued use of statins in patients with hypercholesterolemia causes a deficiency in testosterone and other sex hormones.

**Materials and Methods:**

Systematic Review with Meta-analysis, performed in Embase, Medline and Cochrane databases, until May 2023; PROSPERO CRD42021270424protocol. Selection performed by two independent authors with subsequent conference in stages. Methodology based on PRISMA statement. There were selected comparative studies, prospective cohorts (CP), randomized clinical trials (RCT) and cross-sectional studies (CSS) with comparison of testosterone levels before and after statin administration and between groups. Bias analysis were evaluated with Cochrane Tool, The Newcastle-Ottawa Scale (NOS), and using the Assess the Quality of Cross-sectional studies (AXIS) tool.

**Results:**

There were found on MedLine, Embase and Cochrane, after selected comparative studies, 10CP and 6RCT and 6CSS for the meta-analysis. In the Forrest plot with 6CSS, a correlation between patients with continuous use of statins and a reduction in total testosterone was evidenced with a statistically significant reduction of 55.02ng/dL (95%CI=[39.40,70.64],I²=91%,p<0.00001). In the analysis with 5RCT, a reduction in the mean total testosterone in patients who started continuous statin use was evidenced, with a statistical significance of 13.12ng/dL (95%CI=[1.16,25.08],I²=0%,p=0.03). Furthermore, the analysis of all prospective studies with 15 articles showed a statistically significant reduction in the mean total testosterone of 9.11 ng/dL (95%CI=[0.16,18.06],I²=37%,p=0.04). A reduction in total testosterone has been shown in most studies and in its accumulated analysis after statin use. However, this decrease was not enough to reach levels below normal.

**Conclusion:**

Statins use causes a decrease in total testosterone, not enough to cause a drop below the normal range and also determines increase in FSH levels. No differences were found in LH, Estradiol, SHBG and Free Testosterone analysis.

## INTRODUCTION

Statins are one of the most prescribed medications worldwide for lowering cholesterol. Therefore, they are efficient for the primary and secondary prevention of cardiovascular diseases (CVD) (1, 2). Because cholesterol is one of the precursors of adrenocortical and gonadal hormones, there is a concern that 3-hydroxy-3-methylglutaryl coenzyme A (HMGCoA) reductase inhibitors may impair testosterone production and other sex hormones (3, 4). This could lead eventually to hypogonadism in men. Defined as low levels of total serum testosterone (less than 300 ng / dL) and free testosterone (less than 5 ng / dL) in combination with clinical symptoms such as low sex drive, fracture associated with osteoporosis and erectile dysfunction, or two or more of the following symptoms: sleep disturbances, depressed mood, lethargy, or decreased physical performance (5). The male hypogonadism can thus affect the function of multiple organs and the quality of life of patients.

Conflicting evidence on the subject appears in studies in the medical literature. The study by Bernini GP 1998 evaluated in 8 patients using statins for 24 weeks that there was no change in the testosterone level nor the spermogram (6). The Braamskamp MJ et al. 2015 study evaluated children with familial hypercholesterolemia for 10 years using any statin and compared them with siblings who were not using the medication and found no difference in hormone levels (7). However, in the study by Baspınar O et al. 2016, a correlation was seen between the fall in low-density lipoprotein cholesterol levels in patients using statin with the fall in the levels of total and free testosterone, in addition to exposing an association with the impairment of erectile function assessed by the IIEF-5 questionnaire. Thus, lower cholesterol levels were directly associated with lower testosterone levels and lower IIEF-5 scores (8). Other studies have shown indirect signs of significant hormonal changes, with a drop in PSA in patients without prostate cancer and an increased risk of gynecomastia in men using statins (9, 10). In the cross-sectional study by Stanworth RC et al. 2009, it was not correlated the decrease in testosterone with signs and symptoms of hypogonadism, assessed by ADAM questionnaire, even though it showed a statistically significant reduction in total testosterone and SHBG (11).

Due to the contradictory findings in the literature, the hypothesis of this study is that continuous use of statins may lead to decreased levels of testosterone and other sex hormones in patients with hypercholesterolemia, potentially resulting in hypogonadism. The primary objective is to assess whether continued use of statins in patients with hypercholesterolemia causes a decrease in testosterone levels. The secondary aim is to evaluate the hormonal axis, including free testosterone, estradiol, LH, FSH, and SHBG, with the chronic use of statins.

## METHODS

### Registration and protocol:

PROSPERO CRD42021270424 protocol registration

### Eligibility criteria

Methodology based on the PRISMA 2020 statement (12). Inclusion criteria: Male patients with hypercholesterolemia or dyslipidemia or with cardiac indication for statin use. Intervention: continuous use of any type of statin such as atorvastatin, fluvastatin, lovastatin, rosuvastatin, pravastatin and others. In its various dosages as long as above the established minimum. Comparison: before and after statin use, comparison between control or placebo groups. Outcomes: Hormonal evaluation with total testosterone, free testosterone, FSH, LH, Estradiol, SHBG. Use of a questionnaire to assess sexual function. Study design: Prospective and retrospective comparative studies. Among them are randomized clinical trial (RCT), prospective cohort (PC), cross-sectional study or ecological study (CSS). Search Period: All articles published up to the date of the last search. Language: there was no language restriction. Exclusion Criteria: Patients under 18 years old. Studies that showed divergence between results and measurement units. Articles with incompletely displayed results or not submitted to peer-review journals.

### Information sources

The search was carried out in MEDLINE through PubMed, Embase and Cochrane Central. The review was carried out in all databases in May 2023. Gray searches were carried out by the authors in the references of the selected articles.

### Search strategy

Search strategy performed by author FPAG and revised by LSL. Strategy performed based on PICO acronym (patient, intervention, comparison, and outcome) and study objective using MESH terms. Conducted preliminary search with selection of articles to improve the search with terms found. After performing a definitive search. If during the search any article was found in the gray search that was not included in the search, the search strategy was updated.

Pubmed search strategy: (Testosterone OR androgen OR hypogonadism OR gonadotropin OR Gonadal Steroid Hormones OR Sex Hormone OR Sex Steroid Hormones) AND (CS-514 OR statin OR simvastatin OR atorvastatin OR fluvastatin OR lovastatin OR rosuvastatin OR pravastatin OR 3-hydroxy- methylglutaryl-CoA reductase).

Cochrane search strategy: (Testosterone OR androgen OR hypogonadism OR gonadotropin OR Gonadal Steroid Hormones OR Sex Hormone OR Sex Steroid Hormones) AND (CS-514 OR statin OR simvastatin OR atorvastatin OR fluvastatin OR lovastatin OR rosuvastatin OR pravastatin OR 3-hydroxy- methylglutaryl-CoA reductase).

Embase search strategy: (Testosterone OR androgen OR hypogonadism OR gonadotropin OR Gonadal Steroid Hormones OR Sex Hormone OR Sex Steroid Hormones) in Title Abstract Keyword AND (CS-514 OR statin OR simvastatin OR atorvastatin OR fluvastatin OR lovastatin OR rosuvastatin OR pravastatin OR 3 hydroxy methylglutaryl CoA reductase) in Title Abstract Keyword - in Trials (Word variations have been searched).

### Selection process

The article selection process was carried out in stages in order to screen the articles by double selection. Selection performed from outside paired by two authors in the stages of selection by title, abstract and full text. No automation method was used in the process. Selections were based on eligibility criteria. When an article disagreed, a third author decided.

### Data collection process

Data extraction was also performed by two different authors separately, RSS and FPAG. After extraction, the data were compared with each other, and the PICO table and the results table were created in an excel spreadsheet. Any misunderstanding, a third author resolved, LSL. There was no automation of the process.

Articles that had more than one comparison group were selected, the groups that fit the selection criteria, even if there were more than two selectable groups.

### Data items

The information collected was: Authors, Study year, Study country, Number of patients, Follow-up, Study design, Drug used, Drug dose, Dropouts, Total Testosterone, Free Testosterone, FSH, LH, Estradiol, SHBG, Prolactin and Erectile Dysfunction. Erectile dysfunction and hypogonadism were assessed using validated questionnaires such as *the International Index of Erectile Function* short form (IIEF-5) (13) and *Androgen Deficiency in Aging Male* (ADAM) questionnaire(14), respectively.

In case there was any information exposed in an incomplete way, it was tried to contact the authors of the articles through e-mail. If there was no response, the data was reported as not provided.

### Study risk of bias assessment

To assess the risk assessment of each study, a different questionnaire was used depending on each study design. For the Randomized Clinical Trials, the Cochrane Collaboration’s Tool (15) was used, for the Prospective Cohorts the Newcastle-Ottawa Scale (NOS) (16) and for the Cross-sectional Studies the AXIS tool (Assess the Quality of Cross-sectional studies) (17). Questionnaires were applied independently by two authors in each article, RSS and FPAG.

### Effect measures

Data were extracted in their means and standard deviations. When the data was exposed only in confidence intervals, a conversion of the same type of standard deviation was performed. The measurement units were converted for standardization and possible comparison of variables. Total testosterone and free testosterone were evaluated in ng/dL; FSH and LH in UI/L; Estradiol in pg/mL and SHGB in nmol / L.

### Synthesis methods/ Reporting bias assessment

Review Manager® software, version 5.4 (The Nordic Cochrane Center, The Cochrane Collaboration, Copenhagen, Denmark, 2020). A meta-analysis of continuous variables was used in the reverse variation test, the mean difference (MD) with a 95% confidence interval (CI) was calculated. The results were generated in graphs (18).

To assess heterogeneity, both the graphic of the forest plot and I² were analyzed. When this value was less than 50%, heterogeneity was considered low and acceptable, and the fixed model was used for analysis. When I² was greater than 50%, heterogeneity was considered important. Studies that caused heterogeneity were removed so that further meta-analyses could be conducted to assess the results, a sensitivity test. If there is true heterogeneity, the analysis model will be changed from fixed to random.

An additional analysis was performed, with the MetaDisc software (19), on the results of total and free testosterone in the statistically significant evaluations, to expose the results of the averages of the meta-analyzed groups and not just the difference between the groups. Only the values are exposed and not the graphics.

The presentation of the results was divided according to the different study designs. No other sub-analyses were performed.

### Certainty of evidence

The GRADEpro tool was used to expose the degree of certainty of the evidence of the meta-analyzed and evaluated outcomes (20).

## RESULTS

### Study selection

**Figure 1:**
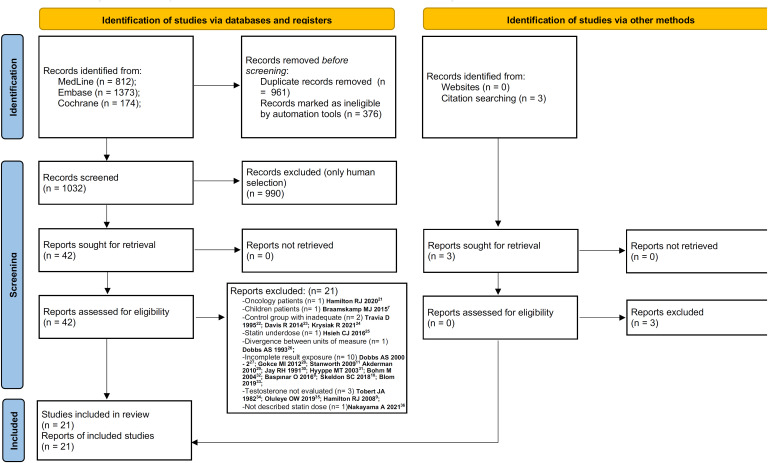
Flowchart of selected articles.

A total of 2359 articles were retrieved in the database searches, of which 812 were from MedLine, 1373 from Embase and 174 from Cochrane. After removing the duplicates, 1032 articles remained, 42 being selected for full reading. Of these, 21 were excluded and 21 selected for systematic review and meta-analysis. The selection flowchart is shown in Figure-1 (7-11, 21-36).

### Study characteristics

The characteristics of the included studies are shown in Table 1. The review included a total of 9879 patients. Selected 21 articles with a total of 9879 patients. Among them, 5 randomized controlled trials (RCT) with 1104 patients, 10 prospective cohorts (PC) with 712 patients and 6 cross-sectional studies (CSS) with 8063 patients.(6, 37-56).

**Table 1:** PC ProspecJve Cohort; RCT- Randomized Clinical Trial; CSS - Cross-secJonal study; SD Standard DeviaJon; Confidence Interval IC; LDL - Low-Density Lipoprotein; DM - Diabetes mellitus; SHA - Systemic Arterial Hypertension; CS - Can’t Say; NA - Not Applicable; FSH - Follicle SJmulaJng Hormone; LH - Luteinizing Hormone; SHBG - sex hormone binding globulin; DHEA - Dehydroepiandrosterone; CV - Cardiovascular; CVD - Cardiovascular Disease;

Study ID		Population			Comparation					Outcomes								
Author Yeay	Study Design	Country	Patient	Age Mean (SD or IC)	Comparison	Drugs and Groups	Dose (mg)	№ patients	Follow Up (Months)	Total Testosterone	Free Testosterone (ng/dL)	FSH (IU/L)	LH (IU/L)	Estradiol (Pg/ml)	SHBG (nmol/L)	DHEA (μg/dL)	Sexual Function Questionnaire	ADAM
**Purvis K 1992^37^**	PC	Norway	Familial Hypercholesterolemia	31(20-49)	Before/After	Simvastatin	40	19/19	3.5	✓	·	✓	✓	·	·	·	·	·
**Bernini GP 1994^38^**	PC	Italy	Mildly Hypercholesterolemic	34 (25 - 57)	Before/After	Simvastatin	10	8/8	6	✓	·	·	·	·	·	·	·	·
**Azzarito C 1996^39^**	PC	Italy	Hypercholesterolemia Ila	56.2 (±2.0)	Before/After	Simvastatin	20	8/8	12	✓	✓	✓	✓	✓	✓	✓	·	·
**Segarra A 1996^40^**	PC	Spain	Hypercholesterolemia in Chronic Kidney Disease	43(±15)	Before/After	Lovastatin	40	25/25	11	✓	·	✓	✓	·	·	·	·	·
**Bernini GP 1998^6^**	PC	Italy	Primary Hypercholesterolemia	48.8 (31-60)	Before/After	Pravastatin	20	8/8	6	✓	·	·	·	✓	·	✓	·	·
**Santini SA 2003^41^**	PC	Italy	Mild To Moderate Hypercholesterolemia and DM	64.7(±7.6)	Before/After	Atorvastatin	20	16/16	3	✓	·			·	✓	✓	·	·
**Dogru MT 2008^42^**	PC	South Africa	Uncontrolled Hyperlipidemia	44.7 (±7.1)	Before/After	Atorvastatin	40	74/74	12	✓	·	·	·	✓	·	✓	IIEF-15	·
**Kocum TH 2008^43^**	PC	Turkey	Men With Arterial Disease Coronary	59 (±9.6) 56 (±11.4)	Before/After and Between Groups	Atorvastatin	20 40	83/83 77/77	12	✓	✓	✓	✓	·	✓		·	·
**Krysiak R 2014^44^**	PC	Poland	Very High Cardiovascular Risk	53.9 (±3.8)	Before/After	Rosuvastatin	20	11/11	4.5	✓	✓	✓	✓	·	✓	✓	·	·
**Krysiak R 2015^45^**	PC	Poland	Coronary Disease After Statin: Increased Aminotransferase Or Creatinokinase	54.3 (±4.0)	Before/After and Between Groups	1- Atorvastatin 2- Rosuvastatin + Ezetimibe	20-40 5-10	12/12 15/15	4	✓	·	✓	✓	·	✓	✓	·	·
**Kan at M 2009^46^**	RCT	Turkey	DM and Coronary Disease Patients	45 (±10)	Before/After and Between Groups	1- Atorvastatin + Ezetimibe 2- Atorvastatin	10+10 80	50/50 48/48	3	✓	·	·	·	✓	·	✓	·	·
**Mastrober ar dino G 1989^47^**	RCT	Italy	Familial Hypercholesterolemia	42.5 (40-45)	Before/After and Between Groups	1- Lovastatin 2- Clofibrate	40 1500	8/8 8/8	1	✓	·	·	·	·	·	·	·	·
**Dobs AS 2000 1^48^**	RCT	USA	Hypercholesterolemia Ila Or llb	41 (±7.3) 41.2 (±6.4) 38.4 (±8.7) 40,2 (±7.5)	Before/After and Between Groups	1- Simvastatin 2- Simvastatin 3- Pravastatin 4- Placebo	20 40 40 CS	37/37 34/34 37/37 30/30	6	✓	✓	✓	✓	·	✓	·	·	·
**Zhi-Guo C 2014^49^**	RCT	China	Elderly Men With Osteopenia And Mild Dyslipidemia	80.8 (±6.8)	Before/After and Between Groups	1- Atorvastatin 2- Lifestyle guidance only	10 NA	32/32 32/32	12	✓	·	·	·	·	·	·	·	·
**Berberoglu Z 2009^50^**	RCT	Turkey	DM with Evident CVD Or CV Risk Factor	60.8 (±7.1) 61.3 (±8.0) 60 (±7.8) 62.2 (±7.5)	Before/After	1-LDL < 70 - Simvastatin 2-LDL < 100 - Simvastatin 3-LDL < 70 - Atorvastatin 4-LDL < 100 - Atorvastatin	35.7 32.7 37.3 34.4	9/9 15/15 10/10 9/9	3	✓	·	·	·	·	·	✓	·	·
**Keyser CE 2015^51^**	CSS	Netherlands	Rotterdam Study Men	64.1 (±8.1) 64.6 (±9.7)	Between Groups	1- Using Statin 2- Non Statin User	CS NA	577 3441	NA	✓	✓	·	·	✓	✓	✓	·	·
**Hall SA 2007^52^**	CSS	USA	USA Population Base	57.9 (±1.3) 45.5 (±0.5)	Between Groups	1- Using Statin 2- Non Statin User	CS NA	237 1575	NA	✓	✓	·	✓		✓	✓	·	·
**Mondul AM 2010^53^**	CSS	USA	USA Population Base	60 42	Between Groups	1- Using Statin 2- Non Statin User	CS NA	41 1275	NA	✓	✓	·	·	✓	·	·	·	·
**Corona G 2010^54^**	CSS	Italy	Men with Sexual Dysfunction	60.9 (±7.6) 60.8 (±7.3)	Between Groups	1- Using Statin 2- Non Statin User	CS NA	244 244	NA	✓	✓	·	·	·	·	·	ANDROTEST	·
**Medras M 2014^55^**	CSS	Poland	Poland Region Population Base	58.6 (±7.6) 57.9 (±5.6)	Between Groups	1- Using Statin 2- Non Statin User	20 NA	38 151	NA	✓	✓	✓	✓	✓	✓	✓	·	·
**Jarari AM 2018^56^**	CSS	Libyan	DM And DM and SHA Men Taking Statin	45.5 (±8.2) 46.8 (±7.1) 44.4 (±5.0) 45.0 (±2.5) 46.5 (±3.1) 44.6 (±4.5) 44.3 (±3.7) 45.9 (±3.8)	Between Groups	1- Non Statin User 2-Using Statin 3-DM Non Statin User 4-DM with Statin <ly 5-DM with Statin >ly 6-DM and SHA Non Statin User 7-DM and SHAwith Statin <ly 8-DM and SHAwith Statin >ly	NA CS NA CS CS NA CS CS	30 30 30 30 30 30 30 30	NA	✓	·	·	·	·	·	·	·	·

### Risk of bias in studies

The risk of bias analysis was assessed using the The Newcastle-Ottawa Scale (NOS), AXIS tool and the Cochrane tool. The risks are shown in supplementary file-1 in appendix.

### Results of syntheses

#### Total Testosterone

**Figure 2:**
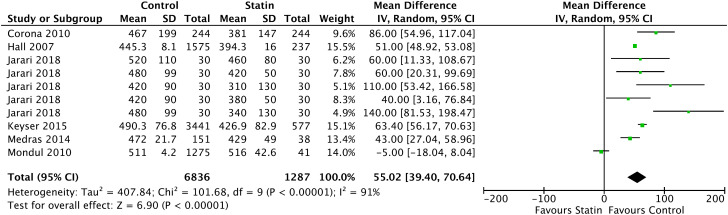
Total testosterone - Cross-sectional studies.

In the Forrest plot with 6 CSS, the correlation between patients with continuous use of statins and reduction in total testosterone was evidenced with a statistically significant reduction between groups of 55.02ng/dL (95% CI = [39.40, 70.64], I2 = 91 %, p < 0.00001), shown in Figure-2 In the continuous statin use group, the mean total testosterone calculated was 409.56ng/dL (95% CI = [384.34, 434.79], p < 0.001) and in the control group, 470.70ng/dL (95% CI = [441.34, 500.05], p < 0.001).

In the analysis with 5 RCTs, there was a reduction in the mean total testosterone in patients who started continuous use of statins, with a statistical significance of 13.12ng/dL (95% CI = [1.16, 25.08], I² = 0%, p=0.03). In the group before statin use, they had a mean testosterone of 411.60ng/dL (95% CI = [335.85, 487.34], p < 0.001) and after the use of 395.14ng/dL (95% CI = [321.38, 468.91], p < 0.001).

**Figure 3:**
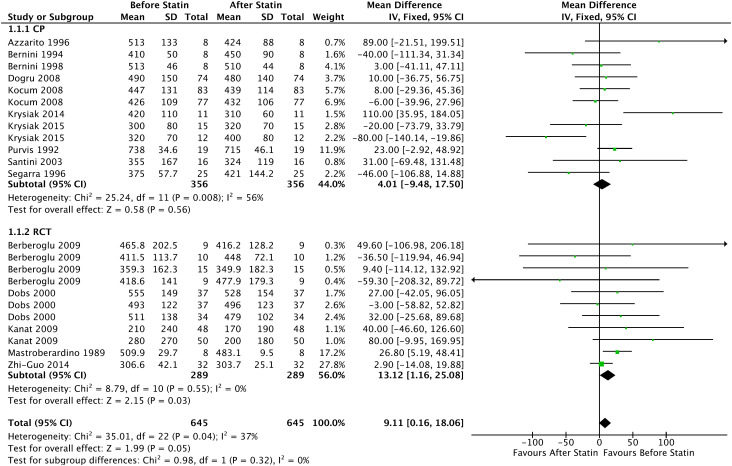
Total testosterone - Before and After - All Prospective Comparative Studies: Prospective Cohort and Randomized Clinical Trial.

Furthermore, analysis of all prospective comparative studies with 15 articles showed a statistically significant reduction in mean total testosterone of 9.11ng/dL (95% CI = [0.16, 18.06], I² = 37%, p = 0.04), shown in Figure-3 In the group before statin use, they had a mean testosterone of 427.83ng/dL (95% CI = [362.25, 493.41], p < 0.001) and after the use of 416.86 ng/dL (95% CI = [365.68, 468.04], p < 0.001).

**Figure 4:**
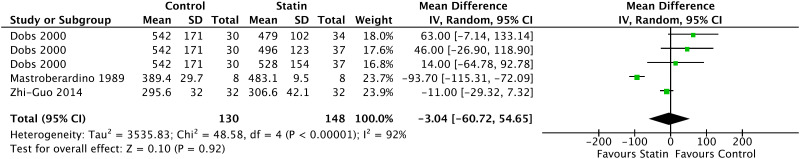
Total Testosterone - Statin X Control - Prospective Cohort.

In the Forrest plot in the analysis with 3 PC, an increase in the mean total testosterone was evidenced, without significant significance, in patients on continuous use of statins and compared with patients in the control group of -3.04 ng/dL (95% CI = [ -60.72, 54.65], I² = 92%, p = 0.92), shown in Figure-4.

#### Free Testosterone

**Figure 5:**
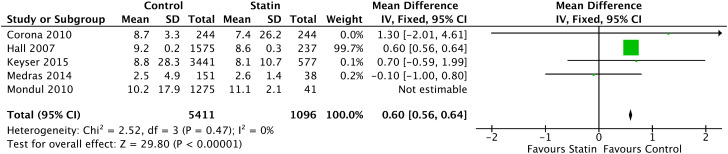
Free Testosterone - Cross-sectional Studies.

In the Forrest plot with 5 CSS, there was a correlation between patients on continuous use of statins and the reduction in free testosterone with a statistically significant reduction of 0.60 ng/dL (95% CI = [0.56, 0.64], I2 = 0%, p<0.00001), shown in Figure-5 In the continuous statin use group, the calculated mean free testosterone was 7.32ng/dL (95% CI = [5.26, 9.38], p < 0.001) and in the control group, 6.64ng/dL (95% CI = [2.88, 10.40], p < 0.001).

**Figure 6:**
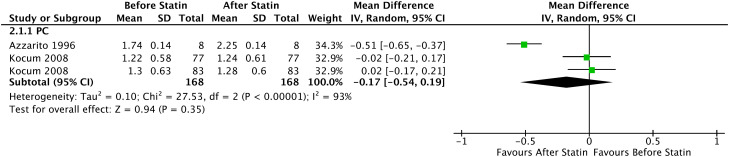
Free Testosterone - Before and After - Prospective Cohort.

In the Forrest plot in the analysis with 2 PC, an increase in the mean of free testosterone in patients who started continuous statin use of -0.17 ng/dL was evidenced (95% CI = [-0.54, 0.19], I² = 93%, p = 0 .35), without statistical significance, shown in Figure-6.

#### FSH

**Figure 7:**
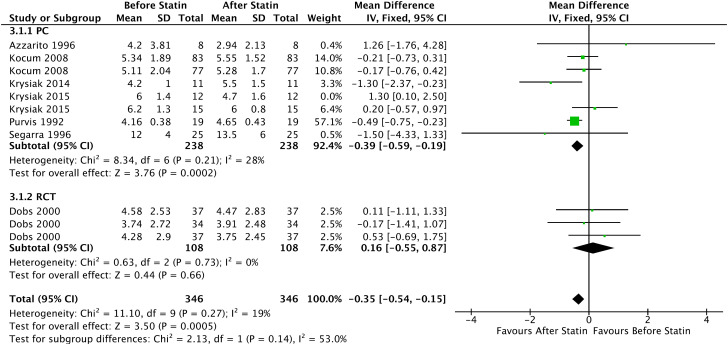
FSH - Before and After - All Prospective Comparative Studies: Prospective Cohort and Randomized Clinical Trial.

The Forrest plot with 6 PC showed an increase in the mean FSH in patients who started continuous statin use of -0.39 UI/L (95% CI = [-0.59, -0.19], I² = 28%, p = 0.0002), with statistical significance. Furthermore, the analysis of all prospective comparative studies with 6 articles showed a statistically significant increase in the mean FSH of -0.35 UI/L (95% CI = [-0.54, -0.15], I² = 19%, p = 0.0005), shown in Figure-7.

#### LH

**Figure 8:**
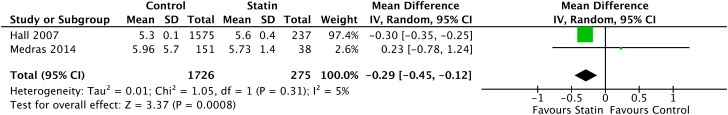
LH - Cross-sectional studies.

In the Forrest plot with 2 CSS, there was evidence of a correlation between patients with continuous statin use and a statistically significant increase in LH of -0.29 UI/L (95% CI = [-0.45, -0.12], I2 = 5%, p <0 .0008), shown in Figure-8.

**Figure 9:**
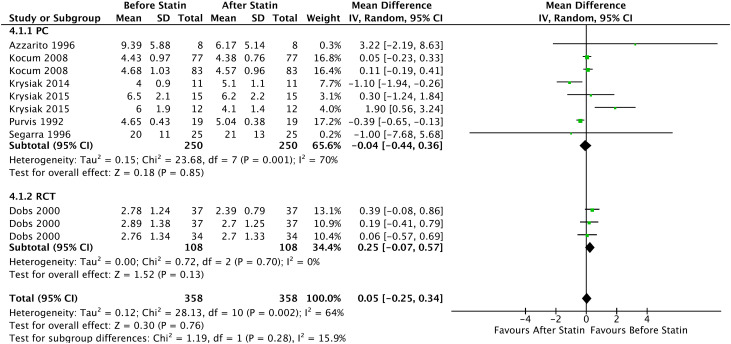
LH - Before and After - All Prospective Comparative Studies: Prospective Cohort and Randomized Clinical Trial.

In the Forrest plot with 5 PC, an increase in the mean LH was evidenced in patients who started continuous statin use of -0.04 UI/L (95% CI = [-0.44, 0.36], I² = 70%, p = 0.85), without statistical significance. Furthermore, in the analysis of all prospective comparative studies with 6 articles, a statistically non-significant reduction in the mean LH of 0.05 UI/L was evidenced (CI 95% = [-0.25, 0.34], I² = 64%, p = 0 .76), shown in Figure-9.

#### Estradiol

**Figure 10:**
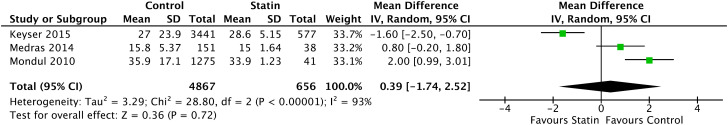
Estradiol - Cross-sectional studies.

In the Forrest plot with 2 CSS, a correlation between patients with continuous use of statins and a decrease in Estradiol without statistical significance of 0.39 pg/mL was evidenced (CI 95% = [-1.74, 2.52], I2 = 93%, p =0. 72), shown in Figure-10.

**Figure 11:**
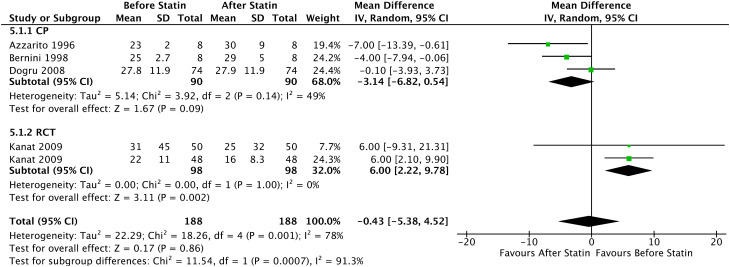
Estradiol - Before and After - All Prospective Comparative Studies: Prospective Cohort and Randomized Clinical Trial.

In the Forrest plot with 3 PC, an increase in the mean estradiol in patients who started continuous statin use of -3.14 pg/mL was evidenced (95% CI = [-6.82, 0.54], I² = 49%, p = 0.09), without statistical significance. Furthermore, the analysis of all prospective comparative studies with 4 articles showed a statistically non-significant increase in the mean estradiol of -0.43 pg/mL (95% CI = [-5.38, 4.52], I² = 78%, p = 0.86), shown in Figure-11.

#### SHBG

**Figure 12:**
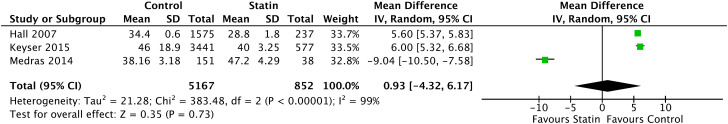
SHBG - Cross-sectional studies.

In the Forrest plot with 3 CSS, there was a correlation between patients with continuous use of statins and a decrease in SHBG without statistical significance of 0.93 nmol/L (95% CI = [-4.32, 6.17], I2 = 99%, p =0, 73), shown in Figure-12.

**Figure 13:**
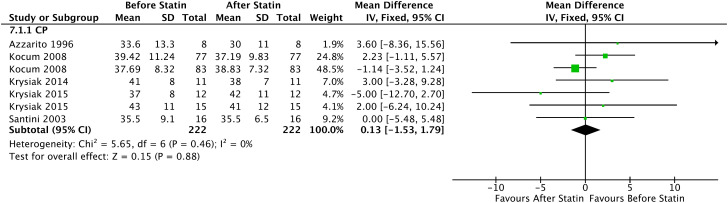
SHBG - Before and After - Prospective Cohort.

In the Forrest plot with 4 PC, a reduction in the mean SHBG in patients who started continuous statin use of 0.13 nmol/L was evidenced (95% CI = [-1.53, 1.79], I² = 0%, p = 0.88), without significance statistics, shown in Figure-13.

#### Certainty of evidence

The summary of evidence and findings are displayed in the GRADE20 table in the supplementary file 2 in appendix.

## DISCUSSION

This study is a comprehensive systematic review and meta-analysis on the subject, which assesses the role of statins use on male hormones, both in the individual and populational context. Thus, in order not to commit any ecological fallacy, it was only accepted as significant evidence, the analyzes that, when there were population studies, had their statistical result in agreement with the prospective studies. In addition, the review included all medications in the statin class used in the articles, without selecting one drug over the others, as previous reviews on the subject did, thus allowing for the effect of the class as a whole. Twenty-one articles were included with a total of 9,879 patients evaluated.

Total Testosterone was seen to decrease its mean at all levels of evidence, with the exception of the comparison between groups in the prospective studies. However, this analysis was hampered due to the low number of articles and patients evaluated, shown in the GRADE evidence summary. Therefore, it is possible to affirm that the statin use causes a decrease in the total levels of testosterone. However, these levels on average did not reach below normality, with the exception of Kannat et al. 2009 data, which were already below normality before starting the medication (46).

There was a decrease in Free Testosterone in the cross-sectional study, but no statistical difference was seen in prospective studies, as there was an important decrease in the number of studies that analyzed the variable. Therefore, it is not possible to state that statin causes a decrease in free testosterone.

Analysis of FSH showed a statistically significant increase in the hormone after statin use. As for the analysis of LH, Estradiol and SHBG, it was not possible to identify statistically significant differences (57).

The limitations of the study were the quality of the data, the mode of exposure of the variables, the variability of the medication, the exposure time and the lack of clinical evaluations. For example, patients with metabolic syndrome and obesity are at risk of testosterone deficiency and usually take statins, and those situations were not evaluated in the studies.

Data quality was a limiting factor, as some articles presented the hormonal outcome as a secondary outcome. In addition, the large variability of data measurement units was one of the possible biases, as it was the cause of the inconsistency of the data in the articles, being a reason for the exclusion of some articles. To homogenize the data, it was necessary to convert units, which generate a limitation and a potential error. For this, the conversion was performed and verified repeatedly by more than one author.

The analysis of several drugs grouped, in different doses and different exposure times can be a potential limiting factor of the evidence, but all the articles included used validated drugs, in their therapeutic dose and with a minimum period of 3 months. Furthermore, the study was unable to establish a correlation between the extent of reduction in total cholesterol levels and the decrease in total testosterone levels. Only a few groups of articles were selected, since not all groups fit the eligibility criteria.

It was not possible to assess sexual function and signs and symptoms of hypogonadism as studies did not assess these data.

Limitations of the human selection process, which include potential selection or analysis errors, were mitigated by employing the methodology recommended by PRISMA, as outlined in the methodology section (12).

Regarding practice implications, the results indicate that statin administration is associated with a decrease in testosterone levels. While this decrease is statistically significant, its clinical relevance may not be substantial. However, in patients at high risk or exhibiting symptoms of hypogonadism or ADAM, statins may contribute to clinical symptoms. Concerning future research directions, there is a necessity for further investigation into the potential relationship between statin use and clinical outcomes such as hypogonadism, ADAM, and erectile dysfunction. To elucidate more accurately the impact of statin or cholesterol reduction on testosterone levels and its clinical consequences, well-designed, multicentric randomized clinical trials are essential. These trials should include control groups of patients using benzofibrates and/or engaging in behavioral modifications like dietary changes and increased physical activity.

## CONCLUSION

Statins use causes a decrease in total testosterone, not enough to cause a drop below the normal range and also determines increase in FSH levels. No differences were found in LH, Estradiol, SHBG and Free Testosterone analysis

## APPENDIX:

**Supplementary File 1:** Risk of bias analysis Table. Assessed using the The Newcastle-Ottawa Scale (NOS), AXIS tool and the Cochrane tool.

Study ID	Selection				Comparability		Outcome		
	Representati veness of exposed cohort (Maximum:★)	Selection of non-exposed cohort (Maximum:★)	Ascertain merit of exposure (Maximum:★)	Demonstration that outcome of interest was not present at start of study (Maximum:★)	Comparability of cohorts on the basis of the design or analysis (Maximum:★★)	Assessment of outcome (Maximum:★)	Follow Up long enough for outcome occur (Maximum:★)	Adequacy of follow up of cohorts (Maximum:★)	Total score (out of 9)
Purvis, et al. 1992(37)	★	★	★	★	★	★	–	★	★★★★★★★(7)
Bernini, et al. 1994(38)	★	★	★	★	★	★	★	★	★★★★★★★★(8)
Azzarito, et al. 1996 (39)	★	★	★	★	★	★	★	★	★★★★★★★★(8)
Segarra, et al. 1996(40)	★	★	★	★	★	★	★	–	★★★★★★★(7)
Bernini, et al. 1998 (6)	★	★	★	★	★	★	★	★	★★★★★★★★(8)
Santini, et al. 2003 (41)	★	★	★	★	★	–	–	★	★★★★★★(6)
Dogru, et al. 2008 (42)	★	★	★	★	★	–	★	★	★★★★★★★(7)
Kocum, et al. 2008(43)	★	★	★	★	★	★	★	★	★★★★★★★★(8)
Krysiak, et al. 2014 (44)	★	★	★	★	★	★	–	★	★★★★★★★(7)
Krysiak, et al. 2015 (45)	★	★	★	★	★	★	–	★	★★★★★★★(7)

**Supplementary File 2:** GRADE20 table is the summary of evidence. **Supplementary File 2. GRADE20 table is the summary of evidence.** **Author(s):** **Question:** Does a statin cause a decrease in Testosterone and other hormones in male patients with hypercholesterolenism? **Setting:** Patients with Hypercholesterolemia **Bibliography:**

Certainty assessment							№ of patients		|Effect		Certainty	Importance
№ of studies	Study design	Risk of bias	Inconsistency	Indirectness	Imprecision	Other considerations	Statin Use		Relative (95% CI)	Absolute (95% Cl)		
**Total Testosterone - Cross-sectional studies. Figure 2.I.**												
6	observational studies	serious^a^	serious^a^	serious^b^	not serious	publication bias strongly suspected strong association all plausible residual confounding would suggest spurious effect, while no effect was observed^c^	6836	1287	–	MD **55.02 ng/dl higher** (39.4 higher to 70.64 higher)		IMPORTANT
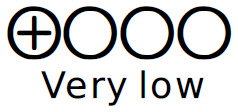
**Total testosterone - Before and After - Prospective Cohort. Figure 2.11.**												
10	observational studies	not serious	not serious	serious^b^	not serious	publication bias strongly suspected^0^	356	356		MD **4.01 ng/dl higher** (9.48 lower to 17.5 higher)		IMPORTANT
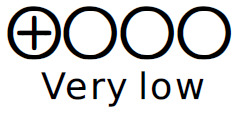
**Total testosterone - Before and After - Randomized Clinical Trial. Figure 2.11.**												
5	randomised trials	not serious	not serious	serious^b^	not serious	none	289	289		MD **13.12 ng/dl higher** (1.16 higher to 25.08 higher)		CRITICAL
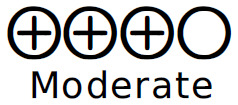
**Total Testosterone - Before and After - All Prospective Comparative Studies. Figure 2.II.**												
15	observational studies	not serious	not serious	serious^b^	not serious	none	645	645		MD **9.11 ng/dl higher** (0.16 higher to 18.06 higher)		CRITICAL
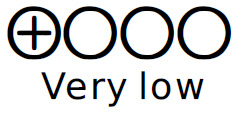
**Total Testosterone - Statin X Control - Prospective Cohort. Figure 2.III.**												
3	randomised trials	not serious	not serious	serious^b^	serious^d^	publication bias strongly suspected^c^	130	148		MD **3.04 ng/dl lower** (60.72 lower to 54.65 higher)		CRITICAL
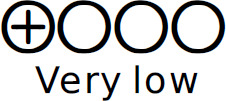
**Free Testosterone - Cross-sectional Studies. Figure 2.1V.**												
4	observational studies	serious^a^	serious^a^	serious^b^	not serious	none	5411	1096		MD **0.6 ng/dl higher** (0.56 higher to 0.64 higher)		IMPORTANT
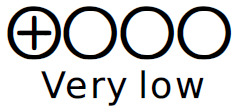
**Free Testosterone - Before and After - Prospective Cohort. Figure 2.V.**												
2	observational studies	not serious	not serious	serious^b^	not serious	publication bias strongly suspected all plausible residual confounding would reduce the demonstrated effect^c,d^	168	168		MD **0.17 ng/dl lower** (0.54 lower to 0.19 higher)		IMPORTANT
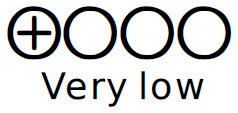
**FSH - Before and After - Prospective Cohort. Figure 2.VI.**												
6	observational studies	not serious	not serious	not serious	not serious	none	238	238		MD **0.39 IU/L lower** (0.59 lower to 0.19 lower)		IMPORTANT
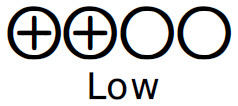
**FSH - Before and After - All Prospective Comparative Studies. Figure 2.VI.**												
7	observational studies	not serious	not serious	not serious	not serious	none	346	346		MD **0.35 IU/L lower** (0.54 lower to 0.15 lower)		IMPORTANT
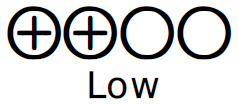
**LH - Cross-sectional studies. Figure 3.I.**												
